# Variation in epibiotic bacteria on two squat lobster species of Munidopsidae

**DOI:** 10.3389/fmicb.2023.1197476

**Published:** 2023-06-28

**Authors:** Wenze Feng, Minxiao Wang, Dong Dong, Min Hui, Huan Zhang, Lulu Fu, Zhaoshan Zhong, Zheng Xu, Chaolun Li

**Affiliations:** ^1^CAS Key Laboratory of Marine Ecology and Environmental Sciences, Institute of Oceanology, Chinese Academy of Sciences, Qingdao, China; ^2^Center of Deep Sea Research, Institute of Oceanology, Chinese Academy of Sciences, Qingdao, China; ^3^University of Chinese Academy of Sciences, Beijing, China; ^4^South China Sea Institute of Oceanology, Chinese Academy of Sciences, Guangzhou, China; ^5^Department of Marine Organism Taxonomy and Phylogeny, Institute of Oceanology, Chinese Academy of Sciences, Qingdao, China; ^6^College of Life Sciences, Zaozhuang University, Zaozhuang, China

**Keywords:** cold seep, squat lobster, epibiotic microbiome, sulfide, adaptation

## Abstract

The relationships between epibiotic bacteria on deep-sea hosts and host lifestyle factors are of particular interest in the field of deep-sea chemoautotrophic environmental adaptations. The squat lobsters *Shinkaia crosnieri* and *Munidopsis verrilli* are both dominant species in cold-seep ecosystems, and they have different distributions and feeding behaviors. These species may have evolved to have distinct epibiotic microbiota. Here, we compared the epibiotic bacterial communities on the *M. verrilli* carapace (MV_carapace_), *S. crosnieri* carapace (SC_carapace_), and *S. crosnieri* ventral plumose setae (SC_setae_). The epibiotic bacteria on SC_setae_ were dense and diverse and had a multi-layer configuration, while those on MV_carapace_ and SC_carapace_ were sparse and had a monolayer configuration. Chemoautotrophic bacteria had the highest relative abundance in all epibiotic bacterial communities. The relative abundance of amplicon sequence variant 3 (ASV3; unknown species in order *Thiotrichales*), which is associated with sulfide oxidation, was significantly higher in SC_setae_ than MV_carapace_ and SC_carapace_*. Thiotrichales* species seemed to be specifically enriched on SC_setae_, potentially due to the synthetic substrate supply, adhesion preference, and host behaviors. We hypothesize that the *S. crosnieri* episymbionts use chemical fluxes near cold seeps more efficiently, thereby supporting the host’s nutrient strategies, resulting in a different distribution of the two species of squat lobster.

## 1. Introduction

Deep-Sea chemoautotrophic ecosystems, including cold seeps, are often characterized by extreme environmental conditions and endemic fauna. Decapod crustaceans, such as alvinocaridid shrimps (Komai et al., 2005), lithodid crabs (Niemann et al., 2013), and squat lobsters (Chevaldonne and Olu, 1996), are common macrofauna in cold seeps. Squat lobsters, as one of the dominant microbenthic crustaceans, are key components of deep-sea chemosynthetic ecosystems, considering their wide distribution and high abundance (Martin and Haney, 2005). Squat lobsters belong to the superfamilies Galatheoidea and Chirostyloidea, with over 1,000 species (Baba and Fujita, 2017). They are found at all depths, and the Munidopsidae (Galatheoidea) species almost completely dominate at extreme depths, with typical adaptations such as eye degeneration (Warrant and Locket, 2004). The Munidopsidae species occupy various ecological niches in the deep sea, including in cold seeps (Martin and Haney, 2005). Most of those observed at cold seeps [such as *Munidopsis andamanica* (Hoyoux et al., 2012) and *Munidopsis* sp. (Macavoy et al., 2008)] have a wide distribution around the vents, with an omnivorous diet. Others [such as *Kiwa puravida* (Thurber et al., 2011) and *Kiwa hirsute* (Macpherson et al., 2005)] are concentrated around vents, with distinctive dense setae on their body surfaces. Numerous invertebrate species have formed symbiotic associations with their microbiomes, which can exert a range of effects on their hosts, predominantly beneficial ones (Mioduchowska et al., 2018). Goffredi et al. proposed that the bacteria that clustered on the plumose setae of *Kiwa hirsute* could be used as a nutrient resource to meet the energetic demands of the host (Goffredi et al., 2008). The provision of nutrients is a major feature of host–microbe interactions (Schwartzman et al., 2015). Dattagupta et al. found that *Niphargus ictus,* a freshwater cave amphipod, developed a nutritional strategy involving scraping epibiotic bacteria (by grooming itself with its gnathopods) to use as food, and it was adapted to environments with high concentrations of sulfides (Dattagupta et al., 2009). Specialized nutritional strategies might have driven the evolution of some Munidopsidae species (Hoyoux et al., 2009). Munidopsidae species’ epibiotic bacterial communities, as a source of nutrients, might be drivers of speciation in squat lobsters.

*Shinkaia crosnieri* (Galatheoidea: Munidopsidae) was first recorded in hydrothermal vents in the Okinawa Trough, mainly dwelling in chemosynthetic ecosystems in the west Pacific Ocean (Baba and Williams, 1998). *Munidopsis verrilli* (Galatheoidea: Munidopsidae) has been widely reported in various deep-sea ecosystems around the world, such as the San Clemente Basin (Williams et al., 2000), Tasmania Island (Baba and Poore, 2002), eastern Hokkaido (Komai and Matsuzaki, 2016), and northeastern South China Sea (Dong and Li, 2015). These two species are closely evolutionarily related and belong to different genera of the same family (Cheng et al., 2020). The plumose setae on *S. crosnieri* (SC_setae_), which harbors dense epibiotic bacterial communities, are the main basis for distinguishing between the two squat lobsters (Wehrtmann and Acuna, 2011). According to a previous study conducted by Watsuji, the host can use the abundant episymbionts that attach to the setae as dense biofilm to obtain nutrients through *in vitro* digestion experiments (Watsuji et al., 2015). Some of these epibiotic bacteria were found to be thioautotrophic based on FISH and Nano-SIMS analysis, as suggested by Watsuji et al. (2012). On the other hand, *M. verrilli*’s underside was smooth without dense plumose setae. Leinberger et al. found evidence for a possible beneficial relationship between *Munidopsis alvisca* and its epibiotic bacteria (Leinberger et al., 2022). However, *Munidopsis* are not generally considered to have a close symbiotic association with their microbiomes but an opportunist. The two species also differ in their distribution in the chemosynthetic ecosystems. *Shinkaia crosnieri* forms impressive aggregations around cold seeps (refs) and hydrothermal vents in the Okinawa Trough (Tsuchida et al., 2003). In contrast, *M. verrilli,* associated with whale carcasses, appears to be an opportunist with a much broader geological range (Williams et al., 2000). It is evident that the concentration of reductive materials is higher in the core regions of vents and seeps, which can lead to higher primary productivity. The distance from the seepages may have influenced the microbiological communities in different squat lobsters. The microbiome’s composition and abundance may, in turn, influence the behavior and evolution of squat lobsters. Thus, we hypothesized that the establishment of symbiosis in SC may contribute to the formation of the current distribution pattern. A systematic comparison of epibiotic bacterial communities associated with different Munidopsidae species would help to explore this hypothesis.

Site F is one of the active cold seeps in the South China Sea and supports several macrobenthic communities, including squat lobsters (Xiao et al., 2020). Both *S. crosnieri* and *M. verrilli* are dominant species in Site F. They have distinct differences in their distribution in relation to the cold seep. Zhao et al. found that *S. crosnieri* only congregated in the immediate vicinity of the cold seep at Site F, with very high abundances, reaching up to 300 individuals per square meter (Zhao et al., 2020). *Munidopsis verrilli,* on the other hand, is distributed over many regions of Site F, but not at high densities (Wang X. et al., 2022). These findings reflect the differential distributions of *S. crosnieri* and *M. verrilli* within a relatively small area. The co-occurrences of the two species provide an excellent opportunity for a comparative study of the link between the epibiotic bacterial communities and the distribution of squat lobsters.

The aim of this study was to investigate the epibiotic bacterial communities on squat lobsters with different ecological niches. We used scanning electron microscopy (SEM) to visualize the morphology of the epibiotic bacteria, quantitative real-time PCR to determine the difference in the absolute density of the bacteria, and high-throughput sequencing of 16S rRNA gene to explore the differences in the composition of the epibiotic bacterial communities.

## 2. Materials and methods

### 2.1. Sample collection

Squat lobsters were collected from Site F in the South China Sea (N119°17′8.22″ E, 22°06′55.26” N) during a cruise in May, 2021. *Shinkaia crosnieri* was caught around the cold seep, where conspicuous methane bubbles can be observed. *Munidopsis verrilli* was mainly caught at peripheral mussel beds around 10 m away from the cold seep. Specimens were collected using a carousel suction sampler on the remotely operated vehicle (ROV) Faxian on the research vessel “Kexue.” They were transported in a homothermic macrofauna carrier which maintained a temperature similar to that at the cold seep, at around 4°C.

All animals were identified as *S. crosnieri* or *M. verrilli via* morphological classification, according to the descriptions given by Baba and Poore (2002) and Baba and Williams (1998). The carapaces of the two squat lobster species (MV_carapace_ and SC_carapace_) and the plumose setae of *S. crosnieri* (SC_setae_) were rinsed with sterilized seawater and then immediately dissected with scissors on board. Samples for molecular analysis were snap-frozen in liquid nitrogen and stored at −80°C until DNA extraction. Cold seep interface water associated with the community of *S. crosnieri* (designated environment [ENV]), which was used to investigate the bacterioplankton, was filtered using an *in-situ* microbe sampler with a 0.22-μm filter membrane and stored at −80°C.

### 2.2. SEM

The animal samples for SEM were preserved in glutaraldehyde, underwent replacement of glutaraldehyde after 24 h, and were then stored at 4°C. In the laboratory, the samples were dehydrated in a graded ethanol series, underwent critical-point drying, were coated with gold (sputter/carbon thread; EM ACE200, Leica, Germany), and were then observed using SEM (S-3400 N, Hitachi, Japan).

### 2.3. Nucleic acid extraction and 16S rRNA gene amplicon sequencing

For 16S amplicon sequencing, we obtained ten different samples. These included three carapace samples of *M. verrilli*, three carapace samples of *S. crosnieri*, three setae samples of *S. crosnieri*, and one environmental water sample for bacterioplankton. We used a DNeasy PowerWater Kit (Qiagen, United States) to extract total DNA from these samples, following the manufacturer’s instructions. The DNA was then diluted to 1 ng/μl for PCR amplification. The PCR amplification was performed using the primer pair 341F (5′-CCTAYGGGRBGCASCAG-3′) and 806R (5′-GGACTACNNGGGTATCTAAT-3′), which target the hypervariable regions between V3 and V4 of the prokaryotic 16S rRNA gene. All PCRs were performed with 10 μl Phusion^®^ HighFidelity PCR Master Mix (NEB, United States), 1 μM forward primer, 1 μM reverse primer, and 1 μL template DNA. The thermal cycling program involved initial denaturation at 98°C for 1 min, 30 cycles of denaturation at 98°C for 10 s, annealing at 50°C for 30 s, and extension at 72°C for 30 s, and a final elongation at 72°C for 5 min. Electrophoresis was then performed using 2% agarose gel. Sequencing libraries were generated using an NEBNext^®^ Ultra™ II DNA Library Prep Kit and were quantified by Qubit and qPCR. After that, NovaSeq 6000 (Illumina) was used for on-machine sequencing.

### 2.4. Quantification of the epibiotic bacteria

Real-time PCR was performed on QuantStudio 1 (Thermo Fisher Scientific, USA) in triplicate. 331F (5′-TCCTACGGGAGGCAGCAGT-3′) and 797R (5′-GGACTACCAGGGTATCTAATCCTGTT-3′) primers were used and each reaction (20 μL) contained 1× SYBR^®^ Premix Ex Taq™ (TaKaRa), 1 μl template DNA, 1 μM forward primer, and 1 μM reverse primer (Nadkarni et al., 2002). The standard curve was constructed based on known amounts of purified PCR product (10^2^ to 10^8^ copies/μl) obtained from *E. coli* genomic DNA by using the bacterial 16S rRNA gene-specific primers 27F (5′-AGAGTT TGATCMTGGCTCAG-3′) and 1492R (5′-TACGGYTACCTTGTTACGACTT-3′) (Weisburg et al., 1991). The thermal cycling program involved 95°C for 15 min, followed by 35 cycles of 95°C for 15 s, 58°C for 30 s, and 72°C for 30 s. The wet weight of each sample was recorded using an electronic balance and the number of copies per unit mass (wet weight) was calculated using the standard curve.

### 2.5. Bioinformatic analyses

Bioinformatic analyses were performed using QIIME 2 (v2022.2) (Bolyen et al., 2019). Raw sequence data were demultiplexed and quality filtered using the q2-demux plugin followed by denoising with DADA2 (Callahan et al., 2016). All amplicon sequence variants (ASVs) were aligned using MAFFT (Katoh and Standley, 2013) and were used to construct a phylogeny using FastTree2 (Price et al., 2010). To minimize differences in sequencing depth among the samples for the alpha and beta diversity analyses, all samples were normalized by subsample at a depth of 32,549. Alpha diversity analysis, beta diversity analysis, and principal coordinate analysis (PCoA) were conducted using q2-diversity. Taxonomy was assigned to ASVs using the q2-feature-classifier (classify-sklearn naïve Bayes taxonomy classifier; Bokulich et al., 2018) and the Silva138 database (Quast et al., 2012). Analysis of variance (ANOVA) and least significant difference (LSD) post-hoc comparisons were applied in SPSS v26 to show the significance of the difference in the alpha diversity index (Shannon index; Fahy et al., 2015). STAMP v2.1.3 was used with Welch’s t-test to compare the dominant bacterial orders and ASVs in different groups (Parks et al., 2014). Analysis of similarities (ANOSIM) based on the Bray–Curtis distance was conducted using the ‘vegan’ package in R v3.5.1 (Liu and Tong, 2017). A bacterial co-occurrence network consisting of the top 200 ASVs was constructed based on a Spearman correlation matrix using the ‘ggClusterNet’ package (Wen et al., 2022).

## 3. Results

### 3.1. Observation of the microbiota on squat lobsters

**Figure 1:**
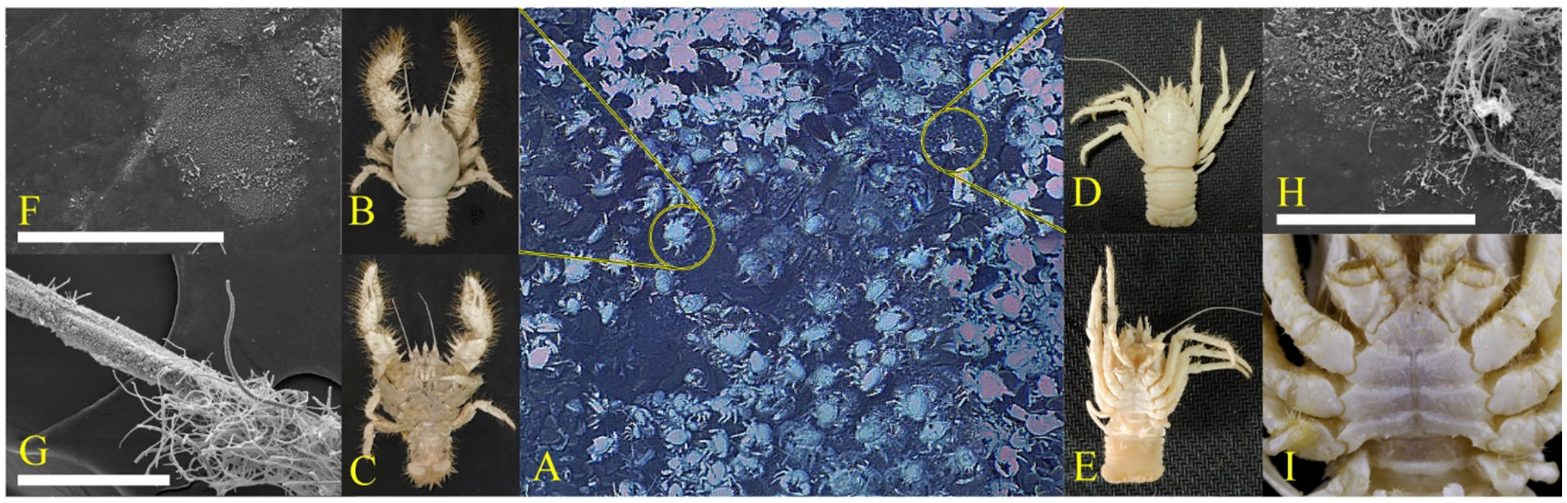
Epibiotic microbiota. **(A)** Photographs of squat lobsters at Site F. The dominant squat lobsters were *Shinkaia crosnieri* **(B,C)** and *Munidopsis verrilli* **(D,E)**. Scanning electron microscopy (SEM) images of the morphology of bacteria attached to the *S. crosnieri* carapace (SC_carapace_) **(F)**, *S. crosnieri* ventral setae (SC_setae_) **(G)**, and *M. verrilli* carapace (MV_carapace_) **(H)**. Dissecting microscope images showing the ventral view of *M. verrilli*, without the setae **(I)**. White scale bar = 100 μm **(F–H)**.

According to Figures 1C,I, the ventral surface of *S. crosnieri* was covered with numerous plumose setae, whereas the ventral parts of *M. verrilli* was much smoother with only nolittle setae. The SEM images showed that the percentage of surfaces covered by biofilm was significantly higher for SC_setae_ (Figure 1G) than SC_carapace_ and MV_carapace_ (Figures 1F,H). In terms of the morphology, the biofilm on the carapace of both species of squat lobster tended to be dominated by groups <5 μm in length and had a monolayer configuration. In contrast, the SC_setae_ epibiotic bacterial community consisted of diverse morphological groups, ranging from 1 to 200 μm in length, with rod or filament shapes, and the community had a multi-layer configuration. In summary, in the setae, the biofilm was much denser and groups with slender linear shapes were more common (Figure 1).

### 3.2. Quantification of the microbiota on squat lobsters

**Figure 2:**
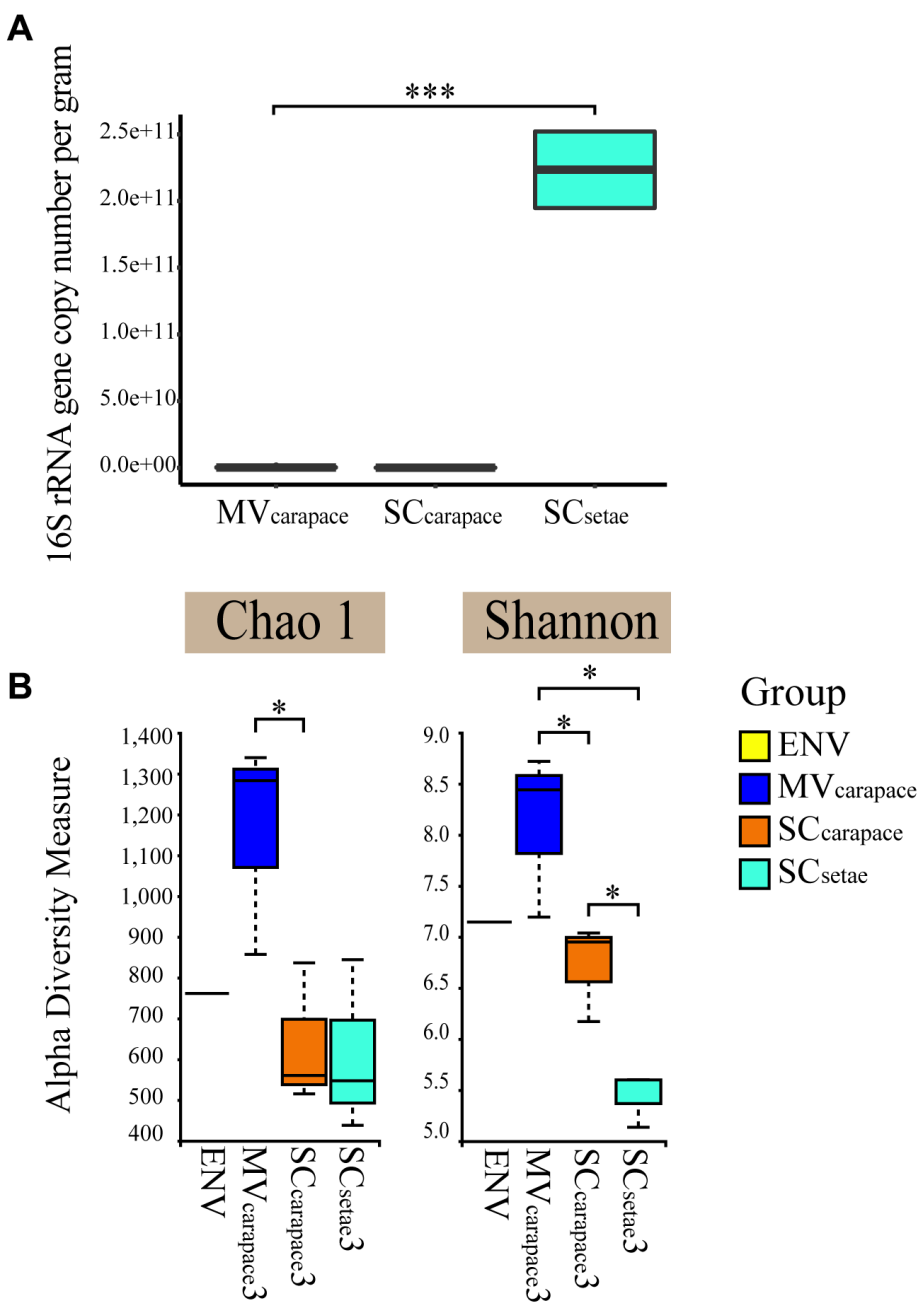
**(A)** Box plots of the 16S rRNA gene copy number of epibiotic bacteria from the *M. verrilli* carapace (MV_carapace_), *S. crosnieri* carapace (SC_carapace_), and *S. crosnieri* setae (SC_setae_). (***: *p* < 0.01). **(B)** Alpha diversity, based on the Shannon index and Chao1 index, of bacterioplankton in cold seep interface water (environment [ENV]) and epibiotic bacteria on *M. verrilli* carapace (MV_carapace_), *S. crosnieri* carapace (SC_carapace_), and *S. crosnieri* setae (SC_setae_). (*: *p* < 0.05).

We established the standard curve with the *R*^2^ of 0.996 to estimate the abundance of the bacteria for each sample (Supplementary Figure S1). Real-time PCR confirmed the significant difference in the 16S rRNA gene copy number of epibiotic bacteria on the two species of squat lobster. The 16S rRNA gene copy number of SC_setae_ exceeded that of MV_carapace_ and SC_carapace_ by around three orders of magnitude, while the values for MV_carapace_ and SC_carapace_ were close. The results suggest that SC_setae_ might provide epibiotic bacteria with an excellent attachment substrate, leading to the enrichment of various groups, with a multi-layer configuration (Figure 2).

### 3.3. Bacterial diversity of different groups

A total of 673,093 reads were generated from the bacterioplankton and epibiotic bacteria associated with MV_carapace_, SC_carapace_, and SC_setae_, and 2,100 ASVs were retained. All sequences found in this study were bacteria due to the use of bacteria-specific primers. The rarefaction curves based on the sequencing data were stable, indicating that sufficient sequencing depth was achieved (Supplementary Figure S2). Based on comparisons with the Silva 138 bacterial database, 52 microbial phyla were detected in the 10 samples, comprising 143 classes, 354 orders, 591 families, and 1,123 genera.

The alpha diversity of the epibiotic bacterial and bacterioplankton communities were compared. The Shannon index varied from 5.13 to 8.68, and differed significantly among the three groups of epibiotic bacteria. The Shannon index was highest for MV_carapace_ and lowest for SC_setae_. And samples at MV_carapace_ showed highest Chao1 indexes. The difference in the epibiotic bacterial communities between carapace and setae was significant (*p* < 0.05). However, the differences between the bacterioplankton (in the single ENV sample) and the epibiotic bacteria were insignificant.

### 3.4. Comparison of bacterial orders and ASVs among groups

**Figure 3:**
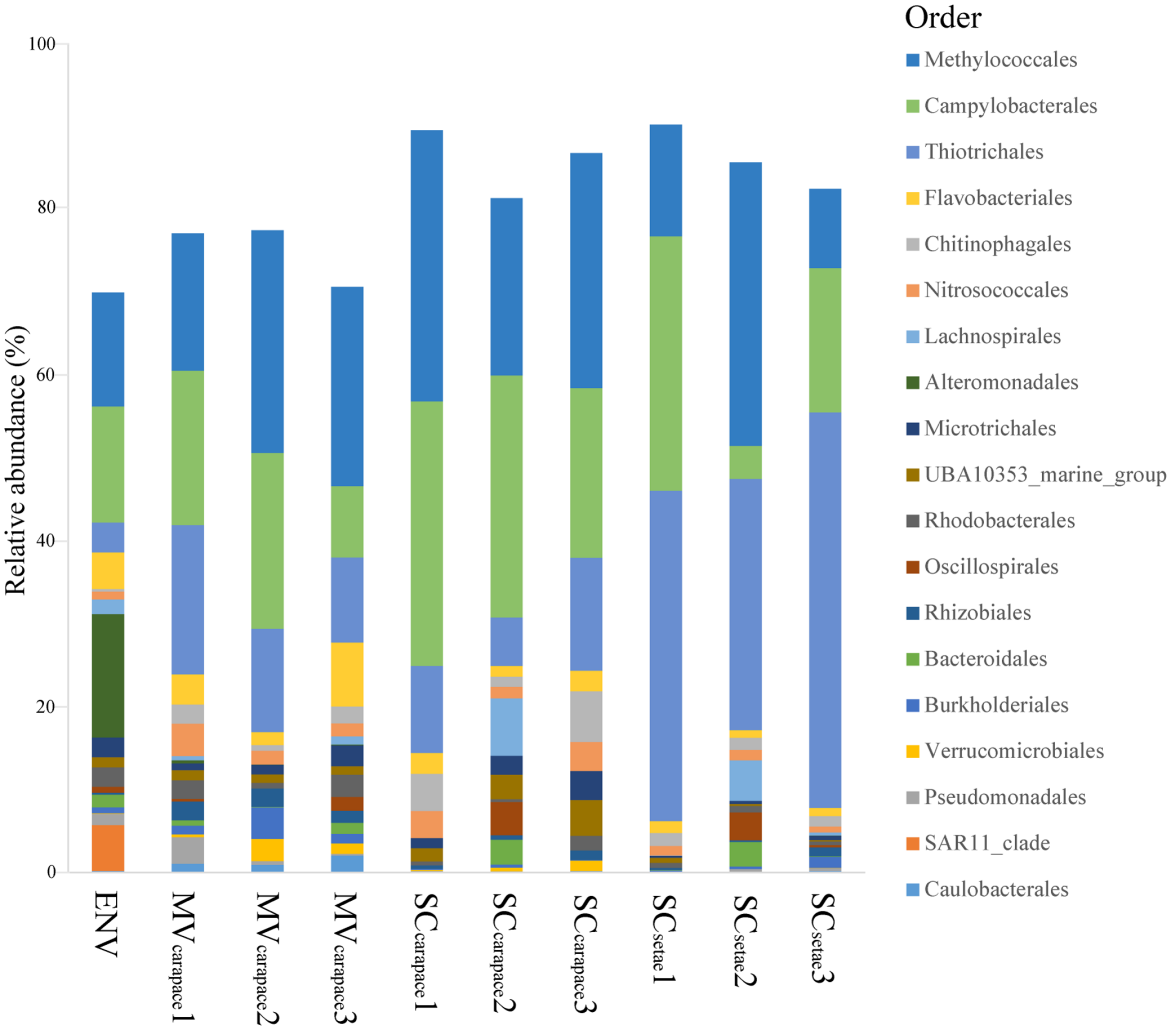
Stacked bar chart of relative abundances of bacterial orders. The top 20 orders in the *M. verrilli* carapace (MV_carapace_), *S. crosnieri* carapace (SC_carapace_), and *S. crosnieri* setae (SC_setae_) samples and the bacterioplankton community in the cold seep interface water (environment [ENV]) sample are shown.

Regarding the three epibiotic bacterial communities, *Methylococcales* (10.25 to 36.75%), *Campylobacterales* (4.32 to 34.32%), and *Thiotrichales* (6.28 to 51.29%) were all in the top three orders in each community, although their ranks varied. In the SC_setae_ epibiotic bacterial communities, *Thiotrichales* accounted for the highest relative abundance (42.25%). In contrast, in the MV_carapace_ and SC_carapace_ epibiotic bacterial communities, the relative abundance of *Thiotrichales* (14.60 and 10.74%, respectively) was lower than that of *Methylococcales* (24.16% and 29.54, respectively) or *Campylobacterales* (17.37 and 29.26%, respectively). Furthermore, the ranking of the top three orders was similar between the epibiotic bacterial communities of MV_carapace_ and SC_carapace_. The bacterioplankton community in the ENV sample wasdominated by *Alteromonadales* (15.99%), *Campylobacterales* (15.03%), and *Methylococcales* (14.77%). Notably, *Alteromonadales* was only exclusively dominated the bacterioplankton community, and the proportion of *Thiotrichales* was much lower (3.87%; Figure 3).

**Figure 4:**
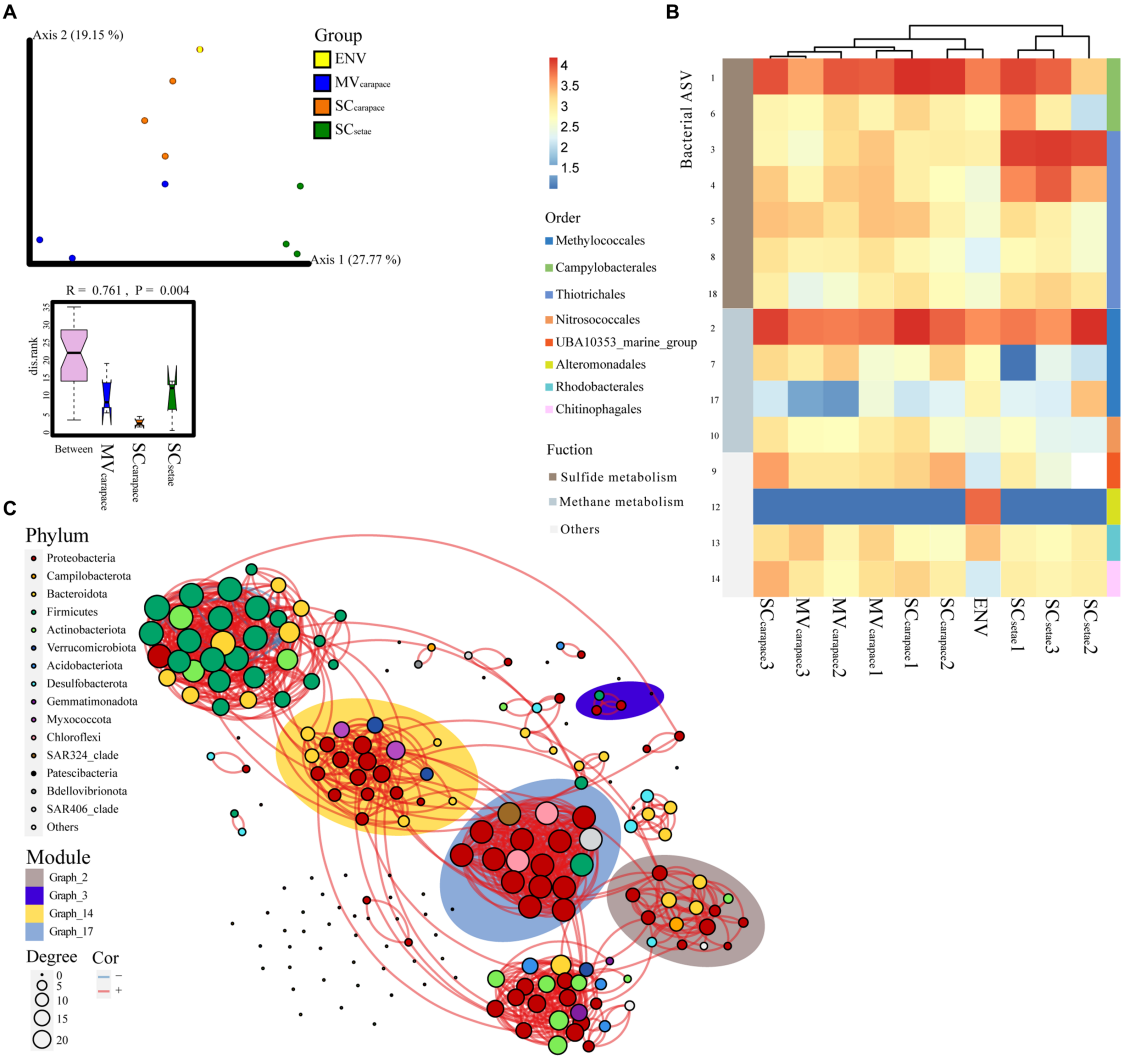
**(A)** PCoA plot of *M. verrilli* carapace (MV_carapace_), *S. crosnieri* carapace (SC_carapace_), and *S. crosnieri* setae (SC_setae_) epibiotic bacterial communities and the bacterioplankton community in cold seep interface water (environment [ENV]), showing the differences in the epibiotic bacterial and bacterioplankton communities at the ASV level. The SC_setae_ samples were clustered far apart from the others. The rank of the dissimilarities within groups of epibiotic bacteria was estimated using analysis of similarity (ANOSIM), revealing significant differences in the epibiotic bacterial communities within each of the three groups. **(B)** Heatmap of the top 10 ASVs associated with sulfur and methane metabolism. **(C)** Bacterial co-occurrence network based on the top 200 ASVs. Each node represents a bacterial ASV. The size of each node is proportional to the number of correlations and the links represent statistically significant correlations (|ρ| > 0.7, *p* < 0.05). The background color around the nodes indicates the modules (groups of tightly connected ASVs), i.e., graph_2, graph_3, graph_14, and graph_17, that the ASVs are affiliated with.

To further explore the compositional differences at the ASV level, PCoA, heatmap analysis, and bacterial co-occurrence network analysis were used (Figure 4). The dominant order *Campylobacterales* mainly involved ASV1 (genus *Sulfurovum*), *Thiotrichales* mainly involved ASV3 (unknown species in order *Thiotrichales*), and *Methylococcales* mainly involved ASV2 (genus Marine_Methylotrophic_Group_2). ANOSIM revealed significant differences in the epibiotic bacterial communities within each of the three groups (*p* < 0.01). The SC_setae_ epibiotic bacterial community was highly separated from the others based on PCoA and heatmap clustering (Figures 4A,B). According to the STAMP analysis, the high abundance of ASV3 (order *Thiotrichales*), associated with sulfide oxidation, in SC_setae_ seemed to be a major source of variation among the groups (Supplementary Figure S3). There was a high degree of similarity between the MV_carapace_ and SC_carapace_ epibiotic bacterial communities, reflected by partial overlaps based on PCoA and heatmap clustering. There were slight differences between individuals in the MV_carapace_ group, as evidenced by the dispersion shown by our results (Figure 4A). The bacterial co-occurrence network, containing 144 nodes and 533 edges, was parsed into various modules, which are groups of tightly connected ASVs. The relative abundance of the module graph_3 (Figure 4C; Supplementary Figure S4), which mainly contained ASV3 (order *Thiotrichales*) associated with sulfur oxidation, was much higher in SC_setae_ than MV_carapace_ and SC_carapace_, which might indicate an enrichment of specific sulfur-oxidizing bacteria in SC_setae_.

## 4. Discussion

### 4.1. Comparison of bacterial orders and ASVs among groups

Cold seeps harbor thriving communities of unique organisms, supported mainly by chemosynthetic microbes (such as *Thiotrichales*, *Methylococcales*, and *Campylobacterales*; Sogin et al., 2021). These microbes produce biomass for the higher trophic levels using energy from reducing gases such as hydrogen sulfide and methane. In this study, we present the first report of the epibiotic bacterial communities associated with *M. verrilli* and the first comparative study of the epibiotic bacterial communities on Munidopsidae species in cold seeps.

Despite the wide distribution of *M. verrilli* in the global deep-sea environment, previous studies on it were limited to morphological identification (Baba and Poore, 2002). Our results showed that chemosynthetic bacteria, including *Thiotrichales*, *Methylococcales*, and *Campylobacterales*, dominated the MV_carapace_ epibiotic bacterial community. *Campylobacterales* species often play a crucial role in the sulfur, nitrogen, and hydrogen cycles, as oxidizers or reducers (Wang Y. et al., 2022). *Methylococcales* belongs to facultative anaerobic bacteria and exhibits prominent capacity for the anaerobic oxidation of methane (Bhattarai et al., 2019). Research on serpulids at a seep known as Jaco Scar showed that *Methylococcales* dominated the epibiotic bacterial communities (Goffredi et al., 2020). Consistent with our results, epibiotic growth of chemoautotrophic bacteria on invertebrates is quite common in cold seeps (Martin and Haney, 2005). Similar to our results on the MV_carapace_ epibiotic bacterial communities, research on *Munidopsis alvisca* showed that chemosynthetic bacterial families were also the dominant families (Leinberger et al., 2022). Research on Alvinocarididae crustaceans at Site F showed that the epibiotic microbes had a similar composition, including *Methylococcales*, *Campylobacterales*, and *Thiotrichales* (Hui et al., 2022). The high relative abundance of chemosynthetic bacteria in MV_carapace_ in our study was in line with the extreme environment involving high hydrogen sulfide and methane concentrations (Cao et al., 2021). Most of the reported mutualistic relationships in deep-sea chemosynthetic ecosystems involve chemosynthetic bacteria with sulfide-oxidizing or methane-oxidizing activity (Levin et al., 2016). Epibiotic microbes have been reported to contribute to the *Munidopsis alvisca* diet, with bacterial biofilms being a relevant food source for *Munidopsis* sp. (Hoyoux et al., 2012). Based on the co-occurrence on *M. verrilli* of methanotrophic and thiotrophic bacteria with high relative abundances, we hypothesize that the epibiotic bacteria might provide nutrition to their hosts, although only a small amount, given their low absolute density.

As for *S. crosnieri,* its epibiotic bacteria have been intensively studied. Watsuji et al. concluded that there is a close relationship between the epibiotic bacteria of *S. crosnieri* and the host in terms of nutrient supply, as the epibiotic bacteria adhering to the setae (including those with sulfide-oxidizing or methane-oxidizing activity) could be scraped by the host to use as food (Watsuji et al., 2017). Our result on the SC_setae_ epibiotic bacterial community composition concurred with previous studies, in which *Thiotrichales*, *Methylococcales*, and *Campylobacterales* made up a large proportion (Xu et al., 2022).

### 4.2. Absence or presence of setae is the main cause of variation in epibiotic bacterial communities

The significant differences in epibiotic bacterial community morphology, density, and composition of SC_setae_ compared to MV_carapace_ and SC_carapace_ hinted at the function of setae in shaping the epibiotic bacterial communities.

The morphology of the dense epibiotic bacteria on *S. crosnieri* that we observed is consistent with previous studies (Zhang et al., 2018). Microbes often live in consortia bound to substrates, forming biofilms, which are quite common in chemosynthetic systems, e.g., euglenozoans in cold seeps are entirely covered by biofilms of closely packed epibiotic bacteria (Buck et al., 2000). Bacterial communities within a biofilm often have a multi-layered structure (Hansen et al., 2007). In our study, the SC_setae_ bacteria, forming a biofilm, were quite different from other epibiotic bacterial communities, with diverse taxa and a multi-layer configuration. A previous study suggested that a multi-layer biofilm configuration involving metabolic cooperation exhibited increased stability and productivity, increasing biomass accumulation due to optimal nutrient utilization (Elias and Banin, 2012). In addition, pereopods densely covered with specialized setae maximized the adhesion space for epibiotic bacteria and the dense structure intensified the adhesion to the surfaces, thereby reducing loss of the bacteria (Christensen et al., 2002; Federle, 2006). Moreover, chemical intermediates produced by reactions within the chemosynthetic microbiota maintained the stability of the biofilm in the face of temporal heterogeneity (Dolinšek et al., 2016). Research has shown that the stable intermediates of the SC_setae_ epibiotic bacterial community play essential roles in metabolic interactions between the hosts and bacteria in the face of temporal oscillations in resource availability (Xu et al., 2022).

The PCoA analysis revealed slight differences in the epibiotic bacteria on the MV_carapace_ and SC_carapace_, which is possibly caused by their different distribution patterns. However, such difference was not supported by the co-occurrence network results and was not visible in the heatmaps, indicating that the microbiome difference is not as remarkable as the difference between the setae and the carapaces. More research is necessary to fully comprehend whether the setae selectively attach to the biofilms of the SC-symbionts. In addition, the overlapping distribution of some individuals of the two squat lobster species around the vents could reduce the significance of the difference between two carapaces in the statistical analysis. The similarity between the MV_carapace_ and SC_carapace_ groups suggested a weak selection of the carapaces by bacteria.

Epibiotic bacterial communities often reflect the environment and can exchange members with the bacterioplankton community in the surrounding water via direct contact (He et al., 2020). A study of Chinese mitten crabs indicated that the bacterial diversity in the water somewhat shaped the epibiotic bacterial communities (Zhang et al., 2016). In our study, almost all the orders present in the bacterioplankton community were present in the epibiotic bacterial community, The presence of plume setae with a soft texture may increase the three-dimensional space for chemosynthetic bacteria to interact with each other and may provide a more suitable adhesion substrate for certain groups. In addition, the extremely low alpha diversity in the SC_setae_ epibiotic bacterial community in contrast to its high density indicates an enrichment of specific bacterial groups.

To identify the enriched taxa, we performed various analyses at the ASV level. Studies have shown that mutualistic host–microbe relationships likely exist for *S. crosnieri* and *M. verrilli* (Watsuji et al., 2012; Leinberger et al., 2022). Additionally, chemosynthetic bacteria played a crucial role in the adaptation of crustaceans to cold seeps by forming close symbiotic relationships with their hosts (Cowart et al., 2017). Therefore, we focused on these chemosynthetic groups in epibiotic bacterial communities to investigate whether these groups were specifically enriched and thus may be involved in differential adaptive strategies. We found that the chemosynthetic orders (*Thiotrichales*, *Methylococcales*, and *Campylobacterales*) accounted for a high proportion of the epibiotic bacterial communities (>60%), while the proportions of these orders were less in the bacterioplankton community (<40%). These differences indicated that the epibiotic bacteria were not random recruited from the bacterioplankton.

The difference in the relative abundance of ASV3 (order *Thiotrichales*) among the epibiotic bacterial communities is striking. *Thiotrichales*, which are often involved in primary production in chemosynthetic communities (Cambon-Bonavita et al., 2020), can exhibit sulfur-oxidizing activity in the presence of hydrogen sulfide and its spontaneously oxidized compounds and has been widely reported in the deep-sea research field, as sulfur-oxidizing symbionts of invertebrate hosts (Hourdez et al., 2021). Dissolved sulfate and elemental sulfur are regarded as indicators of biochemical processes (Feng et al., 2016). Du et al. detected strong Raman peak of the sulfate and elemental sulfur in the fluids in the chemosynthetic communities at Site F (Du et al., 2018). The presence of these chemicals in the vicinity of cold seep could sustain the high biomass production in epibiotic bacterial communities. The relative abundance of ASV3 (order *Thiotrichales*) was strikingly higher for SC_setae_ than MV_carapace_ and SC_carapace_, which might indicate that sulfide oxidation was quite important in the formation of epibiotic bacterial communities on *S. crosnieri* setae.Notably, a recent study found that chemoautotrophic bacteria belonging to Gammaproteobacteria (including the order *Thiotrichales*) could coevolve with their hosts, whereas Campylobacteria (such as the order *Campylobacterales*) had a weak preference for hosts (Lee et al., 2021). This indicates the possibility of enrichment of ASV3 (order *Thiotrichales*) in SC_setae_ and explains the high relative abundance, across all samples, of ASV1 (order *Campylobacterales*), which is also a sulfur-oxidizing bacteria like ASV3 (order *Thiotrichales*).

*S. crosnieri* cluster near the most active cold seeps, where fluids are expelled as a result of geological processes such as sediment compaction (Feng et al., 2018). The chemical fluxes are higher near the seeps with the most active bubble outflows (Sibuet and Olu, 1998). The multi-layer biofilm configuration and high density of the SC_setae_ epibiotic bacterial community helps the host to efficiently utilize the high chemical fluxes around it. Additionally, certain physical behaviors (such as dancing behavior) of the host can indirectly deepen the symbiotic relationship to increase the productivity of its episymbionts and the adaptation of the host, such as the yeti crab *Kiwa puravida* (Thurber et al., 2011). Research suggested that *S. crosnieri* exhibited similar behaviors, allowing it to drive water through the endogenous water flow around its setae (Watsuji et al., 2018). At Site F, there is a vertical gradient regarding the concentration of hydrogen sulfide, with the highest concentration at the bottom interface of the chemosynthetic communities (Cao et al., 2021). Higher concentrations of reducing substances could be produced from the bottom interface due to the endogenous water flow of the hosts. This improves the energy metabolism and the assimilation of chemosynthetic inorganic carbon under the water flow, contributing to an enrichment of sulfur-oxidizing bacteria (such as *Thiotrichales* species) on the setae to support host survival.

As for *M. verrilli*, although the relative abundance of chemoautotrophic bacteria was high, their body surfaces are lacking dense setae, and endogenous water flow and dancing behavior have not been reported or observed. These chemoautotrophic bacteria could not form as dense community as *S. crosnieri*. Previous studies indicated that *M. verrilli* tended to be opportunists and had broader feeding targets (Martin and Haney, 2005). This might indicate that, compared to *S. crosnieri,* a relatively small proportion of the nutrient supply of *M. verrilli* is derived from chemosynthesis by epibiotic microbes utilizing reducing substances (such as sulfide). We hypothesize that the presence of setae shaped the structure of the epibiotic bacterial communities and that the numerous chemosynthetic epibiotic bacteria tightly adhering to SC_setae_ form an ideal biofilm, in contrast to the relatively sparse and simple epibiotic bacterial communities on carapaces, improving metabolic efficiency and the overall primary productivity.

## 5. Conclusion

In light of the unique composition of the SC_setae_ group and the higher relative abundance of ASV3 (order *Thiotrichales*), we suggest that the morphology of *S. crosnieri* might allow the epibiotic bacterial community to form a bushy jungle in the plumose setae, which improves primary production and provides *S. crosnieri* with sufficient nutrition by selectively obtaining sulfur-oxidizing episymbionts from the environment. Consistently, *S. crosnieri* clusters around the vents in order to use the high chemical fluxes for chemosynthetic reactions. In contrast, *M. verrilli* has a relatively low dependence on epibiotic bacterial communities for its nutrient supply, so they are more dispersed. Our research showed that the different distributions of *S. crosnieri* and *M. verrilli* relate to the differences in their epibiotic bacterial communities, involving differential adaptation mechanisms around cold seeps.

## Data availability statement

The data presented in the study are deposited in the NCBI repository, accession number PRJNA947936 and PRJNA954131.

## Ethics statement

The studies involving animals were reviewed and approved by the Science and Technology Ethics Committee of the Institute of Oceanology Chinese Academy of Sciences.

## Author contributions

WF performed the statistical analysis and wrote the first draft of the manuscript. MW and CL contributed to conception and design of the study. ZX, DD, MH, ZZ, HZ, and LF contributed to sampling. All authors contributed to the article and approved the submitted version.

## Funding

This work was supported by the National Natural Science Foundation of China, grant/award number: 42076091 and 41906124, the NSFC Innovative Group Grant (No. 42221005), the National Key R&D Program of China (2022YFC2804003), the Strategic Priority Research Program of the Chinese Academy of Sciences (XDA22050303 and XDB42020401).

## Conflict of interest

The authors declare that the research was conducted in the absence of any commercial or financial relationships that could be construed as a potential conflict of interest.

## Publisher’s note

All claims expressed in this article are solely those of the authors and do not necessarily represent those of their affiliated organizations, or those of the publisher, the editors and the reviewers. Any product that may be evaluated in this article, or claim that may be made by its manufacturer, is not guaranteed or endorsed by the publisher.
