# Supplementary material for: Variation in epibiotic bacteria on two squat lobster species of Munidopsidae
Source: Front Microbiol. 2023 Jun 28;14:1197476. doi: 10.3389/fmicb.2023.1197476 (PMC10336205; doi:10.3389/fmicb.2023.1197476)
Supplement: Supplementary file 1 [file Data_Sheet_1.docx]

Supplementary Material

Variation in epibiotic bacteria on two squat lobster species of Munidopsidae

Wenze Feng^1236^, Minxiao Wang^123^, Dong Dong^4^, Min Hui^4^, Huan Zhang^12^, Lulu Fu^12^, Zhaoshan Zhong^12^, Zheng Xu^5^, Chaolun Li^1236*^

^1^ CAS Key Laboratory of Marine Ecology and Environmental Sciences, Institute of Oceanology, Chinese Academy of Sciences, Qingdao, China

^2^ Center of Deep Sea Research, Institute of Oceanology, Chinese Academy of Sciences, Qingdao, China

^3^University of Chinese Academy of Sciences, Beijing, China

^4^ Department of Marine Organism Taxonomy & Phylogeny, Institute of Oceanology, Chinese Academy of Sciences, Qingdao, China

^5^College of Life Sciences, Zaozhuang University, Zaozhuang, China

^6^ South China Sea Institute of Oceanology, Chinese Academy of Sciences, Guangzhou, China

*** Correspondence:** Chaolun Li: lcl@qdio.ac.cn

# Supplementary Figures and Tables

## Supplementary Figures


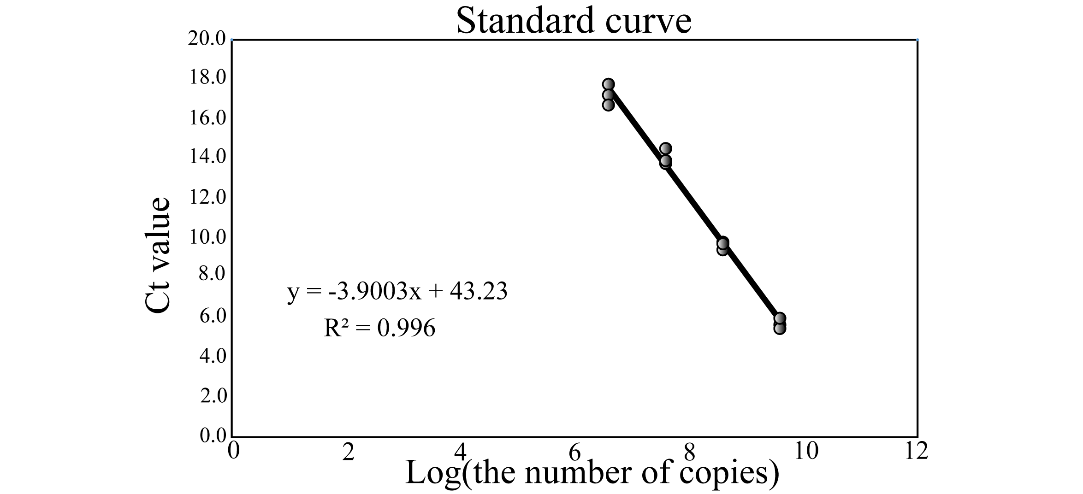


**Supplementary Figure 1.** Standard curves of qPCR.


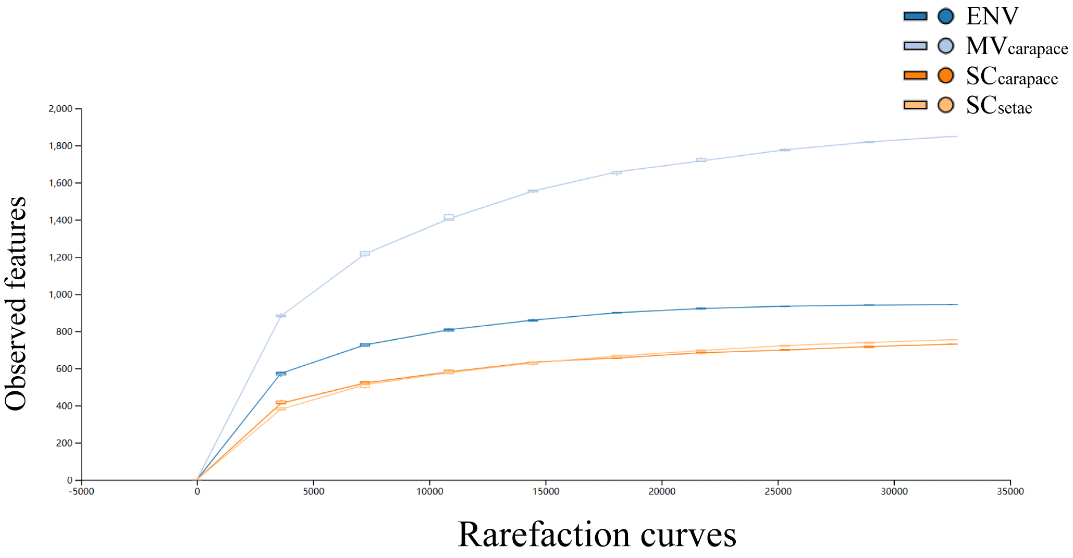


**Supplementary Figure 2.** Rarefaction curves of all the samples


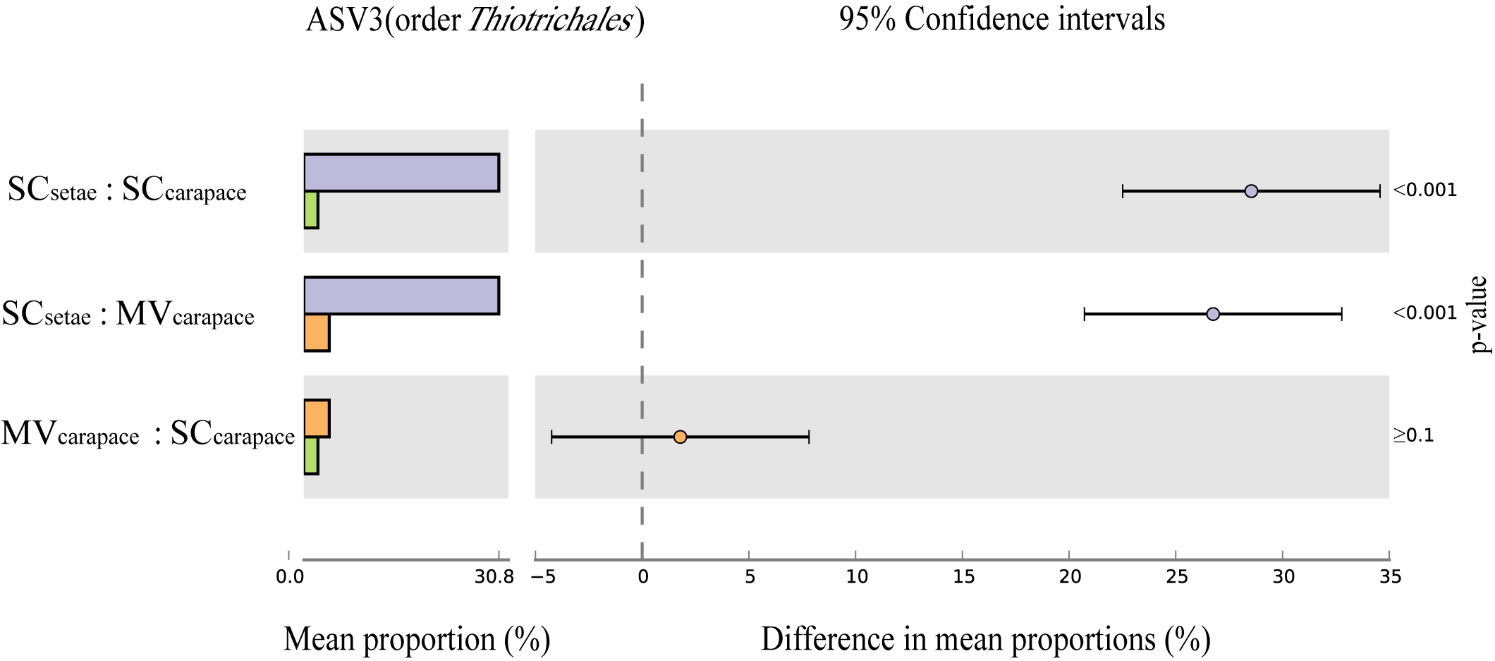


**Supplementary Figure 3.** STAMP analysis showed significant difference in the relative abundance of ASV3 in three epibiotic bacterial communities


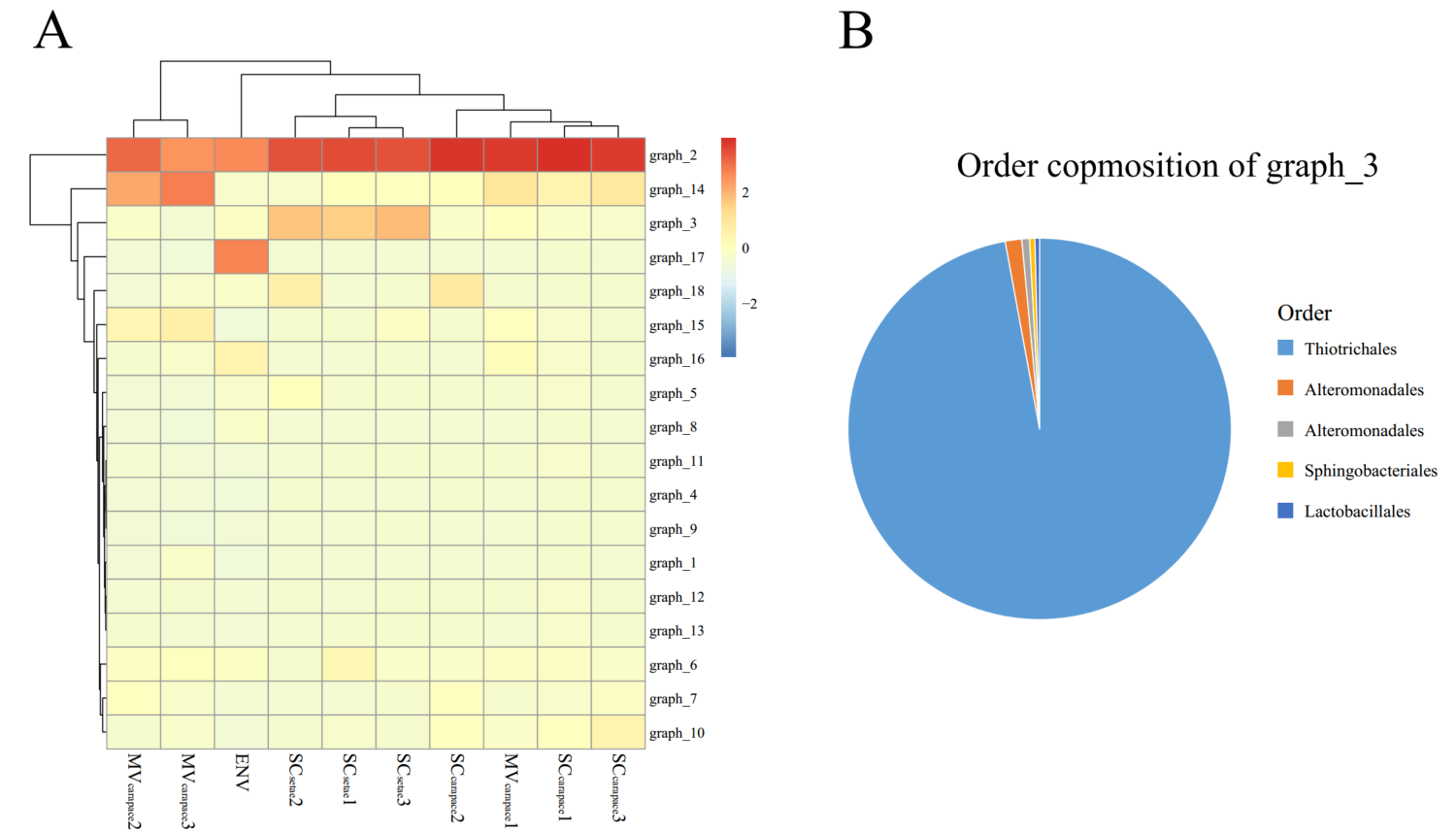


**Supplementary Figure 4.** (A)Heatmap of modules found in microbial interactions network. (B)Composition of the module graph_3 at the order level.
